# Fatal Case of Imported Tick-Borne Encephalitis in South Serbia

**DOI:** 10.3390/tropicalmed7120434

**Published:** 2022-12-13

**Authors:** Lidija Popović Dragonjić, Miodrag Vrbić, Aleksandar Tasić, Verica Simin, Ivana Bogdan, Dragana Mijatović, Alejandro Cabezas-Cruz, Pavle Banović

**Affiliations:** 1 Department for Infectious Diseases, Faculty of Medicine, University of Niš, 18000 Niš, Serbia; 2 University Clinical Center, 18000 Niš, Serbia; 3 Department of Radiology, Faculty of Medicine, University of Niš, 18000 Niš, Serbia; 4 Department for Microbiology, Pasteur Institute Novi Sad, 21000 Novi Sad, Serbia; 5 Ambulance for Lyme Borreliosis and Other Tick-Borne Diseases, Pasteur Institute Novi Sad, 21000 Novi Sad, Serbia; 6 Department for Research & Monitoring of Rabies & Other Zoonoses, Pasteur Institute Novi Sad, 21000 Novi Sad, Serbia; 7 ANSES, INRAE, Ecole Nationale Vétérinaire d’Alfort, UMR BIPAR, Laboratoire de Santé Animale, F-94700 Maisons-Alfort, France; 8 Department of Microbiology with Parasitology and Immunology, Faculty of Medicine in Novi Sad, University of Novi Sad, 21000 Novi Sad, Serbia

**Keywords:** tick-borne encephalitis, tick, Serbia, Switzerland, fatal

## Abstract

Tick-borne encephalitis (TBE) is vaccine-preventable neglected zoonotic neuroinvasive disease, caused by tick-borne encephalitis virus (TBEV). Many of the Central and Eastern European countries are affected by TBE, which is often poorly perceived by tourists visiting endemic territories. Here we are reporting a fatal case of imported TBE in Serbian resident who was exposed to a tick bite during a visit to Switzerland.

## 1. Introduction

Tick-borne encephalitis (TBE) is a neglected zoonotic neuroinvasive disease caused by tick-borne encephalitis virus (TBEV) of the genus *Flavivirus* and family Flaviviridae [1]. TBEV circulates in the natural foci where small mammals act as reservoirs while hard ticks feeding on them serve as vectors and reservoirs. The most important TBEV vector in Europe is the tick *Ixodes ricinus* [2]. Humans are accidental hosts of TBEV and infection occurs when individuals are exposed to the virus through the bite of an infected tick [3]. Human infection can also occur after consumption of contaminated milk or non-pasteurized dairy products, causing-small scale epidemics mostly limited to several households [4].

TBEV foci are complex and dynamic systems requiring specific environment and climate characteristics suitable for the life cycle and/or survival of TBEV vectors and reservoirs, which allow amplification and spreading of the virus. If environmental factors are altered, stable TBEV foci can disappear [5]. New TBEV foci can emerge if the necessary conditions are met and the TBEV transmission chain is completed [6,7].

According to phylogenetic clustering and geographical distribution, five main subtypes of TBEV have been classified as Western (European, TBEV-Eu), Siberian (Eastern, TBEV-Sib), Far-Eastern (TBEV-Fe), Baikalian (TBEV-Bkl) and Himalayan (TBEV-Him). Although TBEV-Eu is the dominant subtype in many European countries, other subtypes (i.e., TBEV-Sib, TBEV-Fe) have been reported in some Baltic countries and in some countries in Eastern Europe as well as Asia [1,3,8].

TBEV endemicity is recognized as a specific problem in Central European countries (i.e., Slovenia, Slovakia, Czech Republic, Austria and Switzerland), where different recommendations related to preventive immunization against TBEV are implemented [3,9]. Depending on the specific country, vaccination against TBEV might be recommended to all residents or only to specific groups identified as high risk to TBEV exposure. Furthermore, TBE is recognized as relevant to travel medicine since tourists visiting endemic territories can be exposed to the TBEV infection either by tick bites during activities in rural environments and/or green-surface areas or by consumption of contaminated milk/dairy products [9,10].

TBE is rarely reported in Serbia and can be regarded as a neglected disease. However, *I. ricinus* ticks infected with TBEV have been detected in several localities of Serbia (e.g., the mountain Fruška Gora, the Belgrade suburban area) [11]. In addition, seroreactivity against TBEV was reported in patients recovered from viral encephalitis of unknown origin [12] and in patients previously infested by ticks [13]. The TBEV infection is most often asymptomatic, but occasionally it can manifest as a fever-like illness and, after an asymptomatic interval, it can progress to complete clinical manifestation (i.e., severe inflammation of the central nervous system (CNS)) [3]. Occasionally, a TBEV infection can be manifested as a febrile illness without CNS involvement, where the febrile state is accompanied by fatigue, headache and myalgia [14]. It is challenging for health practitioners in Serbia to recognize, or suspect, TBEV infection in patients with non-specific viral encephalitis due to the absence of national guidelines with unambiguous TBE case definition [3,12,13].

Here, we report a fatal case of imported TBE in a Serbian resident who was exposed to a tick bite during a visit to Switzerland.

## 2. Case Description

On 26 June 2022, a 58-year-old male with permanent residency in South Serbia was admitted to a local hospital in Northeast Switzerland (the town of St. Gallen: 47.4245° N, 9.3767° E) with quadriparesis, impaired speech, elevated body temperature (up to 38 °C), headache, malaise, muscle pain and diarrhea. He was visiting his family in St. Gallen for 30 days and recalled having a tick bite 17 days prior to hospital admission, while hiking in a forested area near St. Gallen (9 June 2022). He removed the tick by himself using his fingers and discarded it afterwards. He declared no comorbidity, as well as a lack of immunization against TBEV.

The first signs and symptoms started approximately 7 days after the tick bite (16 June 2022), with development of pain in the lower back, headache, malaise and elevation of body temperature. Seven days later, the patient started to feel weakness in his upper and lower limbs and experienced difficulties in speaking and swallowing. His signs and symptoms progressed until he developed breathing difficulties and was not able to talk, swallow, stand or walk. On the 10th day of illness, he was admitted to the hospital, where severe right-sided sensory and motor tetraparesis and hyporeflexia were observed. Meningeal signs were absent, while swallowing, corneal and coughing reflexes were preserved. Immediately after hospitalization, the patient was intubated and mechanically ventilated due to respiratory insufficiency.

Initial laboratory findings on the 14th day of illness revealed mild blood leukocytosis with lymphopenia and clear cerebrospinal fluid (CSF) with mixed pleocytosis (187 cells/μL: lymphocytes 25%, monocytes 21%), a slightly elevated lactate value of 2.6 mmol/L (˂2.4 mmol/L), a slightly elevated protein level (0.74 g/L, albumin 0.4 g/L) and a regular glucose level (3.4 mmol/L). The CSF and serum were negative for oligoclonal bands. The CSF bacterial culture was negative. Infections caused by the herpes simplex virus (1 and 2), varicella-zoster virus, human immunodeficiency virus, SARS-CoV-2 and *Borrelia burgdorferi* sensu lato (s.l.) complex and the existence of autoimmune and paraneoplastic encephalitis were ruled out following negative laboratory findings. IgM reactive to the TBEV antigen were found in serum and CSF, suggesting flaviviral etiology. The complete list of results of the laboratory examination conducted after admission to the hospital is presented in Table 1.

**Figure 1:**
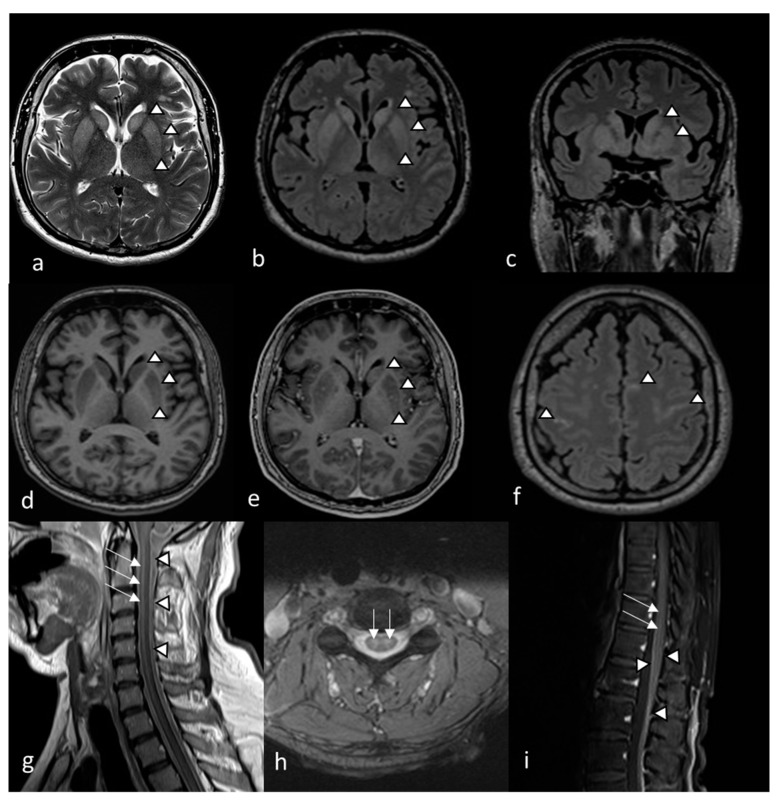
MRI findings on the 14th day of illness in a patient with TBE. The MRI showed symmetric diffuse T2wi (**a**) and FLAIR (**b**). Hyperintensity of the basal ganglia, especially the putamen and caudate nucleus, with a slightly less pronounced increase in the signal of both thalami (arrowheads) on axial (**a**,**b**) and coronal (**c**) images, consistent with edema. Bilaterally decreased T1wi signal in the putamen and caudate nucleus (**d**) followed by patchy contrast enhancement (**e**) on axial images (arrowheads), implying basal ganglia inflammatory lesions. Meningeal contrast enhancement on FLAIR (**f**) and leptomeningeal contrast enhancement in the cervical medulla (**g**) and medullary cone (**i**), correlating with meningeal/leptomeningeal inflammation (arrowheads). Symmetric T2wi signal increase found in cervical medulla (**h**) with contrast enhanced areas delineated in cervical (**g**). Lumbar spinal cord (**i**) correlating with myelitis (arrows).

On the same day, magnetic resonance imaging (MRI) was performed and global diffusion signal interference of the grey substance was detected, indicating encephalitis and long-segment myelitis (Figure 1).

Five days later (the 19th day of illness), the patient was reallocated via helicopter from Switzerland to the Clinical Center Niš (Serbia) for future treatment. Combined antiedematous (mannitol solution), corticosteroid (dexamethasone), anticoagulant, physical and nutritive therapy were initiated. Eventually, the patient gained consciousness and was able to understand and execute orders to open and close his eyes. Status related to quadriplegia, dysphagia and aphasia was unchanged. Subfebrility persisted as it had during hospitalization in Switzerland. The electromyoneurography finding was normal (i.e., no signs of axonopathy, conduction block or demyelination were found). The MRI did not show any differences in the status described on Day 14. The complete list of results of the laboratory examination conducted on Day 19 is presented in Table 1.

On the 27th day of illness, cytology and biochemical findings in CSF were unremarkable except for detection of elevated protein levels (0.92 g/L). The CSF and serum samples were forwarded to the Pasteur Institute Novi Sad for detection of TBEV neutralizing antibodies (TBEV-Nab). See the Supplement File S1 for details concerning the microneutralization assay. Both samples showed a high neutralizing effect, suggesting acute TBEV infection. The complete list of results of the laboratory examination conducted on Day 27 is presented in Table 1.

In the following period, the patient showed no neurological improvement, with the same degree of quadriparesis and a return to the state of disturbed consciousness. The further course of the disease was complicated by ventilator-associated pneumonia, which lead to the fatal outcome on Day 60.

## 3. Discussions

According to European Centre for Disease Prevention and Control (ECDC) reports, the majority of TBE cases in Europe are occurring from May to November. The incidence shows a bimodal distribution with peaks in June–August and October [15]. The case described here is in accordance with current TBE incidence trends as the first signs of disease and nervous system involvement occurred in June. It should be highlighted that the tick bite in our patient occurred during forest hiking, which is a high-risk activity for TBEV exposure in endemic territories, such as Switzerland [8]. For the same reason, pre-exposure immunization against TBE is recommended for travelers visiting endemic countries [3,8,9]. Similar recommendation exists in Serbia, but up to today neither of the two vaccines available in Western Europe (i.e., Encepur and FSME-IMMUN) are registered in Serbia for routine application [3].

In the majority of exposed individuals, TBEV infection is asymptomatic. In approximately 30% of TBEV infection cases, patients will develop fever and flu-like symptoms after an average incubation period of 7 days (with a range of 2–28 days) and 20–30% of those will progress to complete clinical manifestation of TBE. According to anamnestic data related to tick-bite exposure, the incubation period (i.e., the period between tick bite and fever onset) in the case reported here was 7 days and it is in accordance with previously published data [16].

If complete clinical manifestation of TBE occur, CNS involvement is most often presented as a pronounced meningeal syndrome, altered state of consciousness, cognitive dysfunction, ataxia, rigidity, convulsions, tremors, paralysis of cranial nerves and limb paresis [17,18]. Our patient developed complete clinical manifestation of TBEV infection in the form of encephalomyelitis. In fewer than 10% of TBE cases, viral infection affects the anterior horn of the spinal cord which manifests as flaccid poliomyelitis-like paralysis or/and as paralysis of respiratory muscles, requiring artificial ventilation [19,20].

Concerning the virulence of different TBEV subtypes, TBEV-Sib and TBEV-Fe infection usually cause TBE with a higher mortality rate compared to TBEV-Eu where fatal cases occur in 0.5–2% of cases. In the case described here, the patient developed a severe neurological form of the disease after a tick bite within Central European territory, suggesting possible (although not proven) exposure to subtype TBEV-Eu.

A possible reason for the severe manifestation of TBEV infection in this case may be the patient’s age, given that severe disease form and unfavorable outcome of TBE are associated with older age and male gender [17,21]. In addition, an earlier study in tick infested patients from Serbia showed that men have a higher risk of exposure to TBEV than women [13].

The results of slightly elevated leukocyte count, neutrophilia and decreased lymphocytes found here are in accordance with previously reported results [22]. The CRP levels were within reference values and did not reflect the patient status severity. The progression of aspartate aminotransferase, alanine aminotransferase, gamma glutamyl transferase and alkaline phosphatase values throughout the second phase can be explained with maintenance of viremia, suggesting an effect of the virus on the bone marrow and liver [14]. Elevated CK levels could be a consequence of muscle tissue affection presented as myositis, which is shown by some authors as a prognostic sign for a severe clinical course [23]. The results of CSF analysis in our case are in accordance with other TBE cases, since elevated levels of neutrophils, lymphocytes and proteins were detected [22].

In addition to laboratory diagnostics, several imaging methods can be used to assess disease severity and the degree of neurological involvement. The TBEV has tropism for cells of the basal ganglia and the thalamus [18]. It is considered for CT to have lower sensitivity in the diagnosis of TBE compared to MRI, probably due to improper delineation of involved areas, leading to underestimation of the extent of encephalitis. Typical TBE changes include hypodense regions mainly in the basal ganglia and the thalamus [18]. On the other hand, MRI abnormalities can be found in 20–44% of acute TBE cases [17,18,19].

The MRI findings of our patient are very significant due to the simultaneous involvement of meninges, basal ganglia (putamen and caudate nucleus), thalamus (bilaterally), meninges, and a cervical and lumbar spinal cord without significant improvement throughout care in the control scan. The extent and the dynamics of the process explained the progressive and severe clinical presentation and the poor outcome of the disease. The increased extension of cerebral MRI lesions in TBE patients is associated with a more severe clinical course [24].

Given that there are no available anti-TBE vaccines in Serbia, the case described here stresses the necessity of introducing immunization against TBEV in persons traveling from Serbia to TBE-endemic territories (e.g., Croatia, Slovenia, Hungary, Slovenia, Slovakia, Czech Republic and Switzerland, among others). Considering previously published data suggested exposure of the Serbian population to TBEV [11,12,13], implementation of immunization would allow prevention of autochthonous TBE in persons at risk, as well as prevention of imported TBE cases.

## Figures and Tables

**Table 1:** Laboratory findings in patient with TBE.

Parameter of Interest	Day of Disease			Ref. Values
	14	19	27	
**Biochemical and Hematological Analyses**				
Leukocyte count (10^9^/L)	11.3	7.5	12.2	4.0–10.0
Neutrophils (%)	86.2		78.7	25.0–78.0
Lymphocytes (%)	6.6		10.2	20.0–52.0
Creatine kinase (U/L)	496			<170
C-reactive protein (mg/L)	7		6.4	<8
Glucose (mmol/L)	6.1		7.9	3.9–5.6
Aspartate aminotransferase (U/L)	41	413		<55
Alanine aminotransferase (U/L)	16	557	429	<55
Gamma-glutamyl transferase (U/L)	55	1025		<65
Alkaline phosphatase (U/L)	78	359	434	30–120
**CSF Analyses**				
Leukocytes (per mm^3^)	187		0	<5
Neutrophiles (%)	55		0	0
Lymphocytes (%)	25		0	0
Monocytes (%)	21		0	0
Protein (g/L)	0.74		0.92	<0.45
Albumin (g/L)	0.4			<0.38
Glucose (mmol/L)	3.4		3.9	2.5–4.4
**Microbiological Analyses**				
SARS-CoV2 Ag	NEG.			NEG.
HIV 1&2 Ab/Ag	NEG.			NEG.
Treponema pallidum Ig	NEG.			NEG.
TBEV IgM (U/mL)	10			<10
TBEV IgG (U/mL)	<30			<100
TBEV IgM CSF-S Index	>2.49			<1.5
TBEV IgG CSF-S Index	<1.5			<1.5
TBEV NT50 CSF			640	≤10
TBEV NT50 S			1280	≤10
B. burgdorferi IgG S (AU/mL)	9			<10
B. burgdorferi IgM S (AU/mL)	6			<18
HSV1 DNA (CSF)	NEG.			NEG.
VZV DNA (CSF)	NEG.			NEG.
WNV RNA (CSF)	NEG.			NEG.
**Autoimmune Encephalitis Panel CSF**				
Aquaporin/MOG	NEG.			NEG.
NMDA–Receptor IgG	NEG.			NEG.
VGKC	NEG.			NEG.

CSF—cerebrospinal fluid, HIV—Human immunodeficiency virus, Ag—antigen, Ig—immunoglobulin, S—serum, HSV1—herpes simplex virus 1, VZV—varicella zoster virus, WNV—West Nile virus, MOG—myelin oligodendrocyte glycoprotein, NMDA—N-methyl-D-aspartate, VGKC—Voltage-gated potassium channel.

## Data Availability

Not applicable.

## Supplementary Materials

The following supporting information can be downloaded at: https://www.mdpi.com/article/10.3390/tropicalmed7120434/s1, File S1–Microneutralization assay procedure.
