# Supplementary material for: Fatal Case of Imported Tick-Borne Encephalitis in South Serbia
Source: Trop Med Infect Dis. 2022 Dec 13;7(12):434. doi: 10.3390/tropicalmed7120434 (PMC9784870; doi:10.3390/tropicalmed7120434)
Supplement: Supplementary file 1 [file tropicalmed-07-00434-s001.zip › tropicalmed-2059980-supplementary.pdf]

Tropical Medicine and Infectious Disease

Supplementary Materials: Microneutralization assay (micro-NT) procedure

Lidija Popović Dragonjić [1,2,*], Miodrag Vrbić [1,2], Aleksandar Tasić [2,3], Verica Simin [4], Ivana Bogdan [4], Dragana Mijatović [5,6], Alejandro Cabezas-Cruz [7] and Pavle Banović [5,8,*]

1 Department for Infectious Diseases, Faculty of Medicine, University of Niš, Niš 18000
2 University Clinical Center, Niš, Niš 18000, Serbia
3 Department of Radiology, Faculty of Medicine, University of Niš, Niš 18000, Serbia
4 Department for Microbiology, Pasteur Institute Novi Sad, Novi Sad 21000, Serbia
5 Ambulance for Lyme Borreliosis and Other Tick-Borne Diseases, Pasteur Institute Novi Sad, Novi Sad 21000, Serbia
6 Department for Research & Monitoring of Rabies & Other Zoonoses, Pasteur Institute Novi Sad, 21000 Novi Sad, Serbia
7 ANSES, INRAE, Ecole Nationale Vétérinaire d'Alfort, UMR BIPAR, Laboratoire de Santé Animale, Maisons-Alfort, F-94700, France
8 Department of Microbiology with Parasitology and Immunology, Faculty of Medicine in Novi Sad, University of Novi Sad, Novi Sad 21000, Serbia
* Correspondence: lidija_popovic2003@yahoo.com (L.P.D.); pavle.banovic@mf.uns.ac.rs (P.B.)

Citation: Popović Dragonjić, L.; Vrbić, M.; Tasić, A.; Simin, V.; Bogdan, I.; Mijatović, D.; Cabezas-Cruz, A.; Banović, P. Fatal Case of Imported Tick-Borne Encephalitis in South Serbia. *Trop. Med. Infect. Dis.* **2022**, *7*, x. https://doi.org/10.3390/xxxxx

Academic Editor(s): Harunor Rashid

Received: 15 November 2022
Accepted: 9 December 2022
Published: date

Publisher's Note: MDPI stays neutral with regard to jurisdictional claims in published maps and institutional affiliations.

Copyright: © 2022 by the authors. Submitted for possible open access publication under the terms and conditions of the Creative Commons Attribution (CC BY) license (https://creativecommons.org/licenses/by/4.0/).

TBEV strain Neudörfl (National Collection of Pathogenic Viruses, United Kingdom; Cat. No 0201139v) was cultured in monolayer of BHK-21/C13 (BS CL 8, Istituto Zooprofilattico Sperimentale Brescia, Italy) in BSL2+ laboratory of Pasteur Institute Novi Sad. Virus stocks were prepared in concentration of 100 Tissue Culture Infectious Dose (TCID)/100 µl and stored at -80° C until further use.

The micro-NT was performed in 96-well cell culture plate (Thermo Scientific™ , Massachusetts, United States, Cat. no 130338). After sample inactivation at 56 ^{o}C for 30 min, serum and cerebrospinal fluid samples were tested in duplicate, diluted in Glasgow Minimal Essential Medium (Biowest, France; Cat. No P0120) in serial dilutions of 1:5 to 1:640.

In every test run, defined positive and negative control were added together with a cell control and a virus back-titration. A total of 100 TCID of virus stock was added to the respective serum dilutions and incubated for one hour at 37 ^{o}C. Subsequently, serum-virus/CSF-virus mixture was transferred to wells with previously seeded BHK21/C13 $2x10^4$ cells and incubated for five days at 37 ^{o}C in atmosphere with 5% CO2. For each sample, cytopathic effect (CPE) in both wells was observed (Figure 1). The sample dilution resulting in virus neutralization in 50% of the replicates (NT50) was calculated using the method of Spearman and Karber. Serum/CSF sample with ≥ 1:10 NT50 for neutralization assay was interpreted as a positive result.

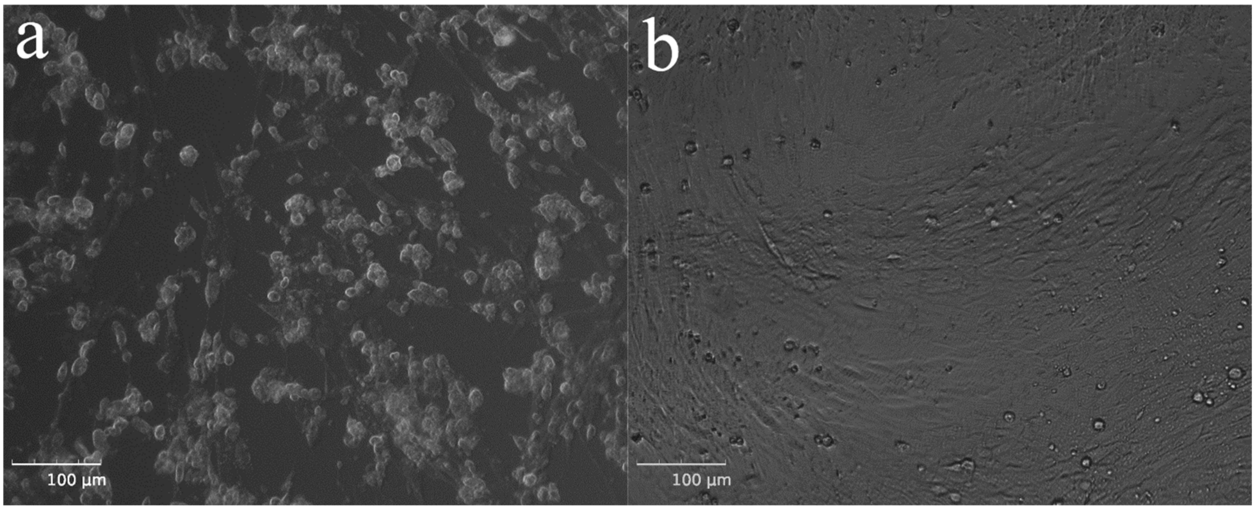

Figure S1. Micro-NT assay results. a) CPE detected in BHK-21/C13 cell line infected with TBEV; b) Absence of CPE in BHK-21/C13 cell line monolayer due to virus neutralization.
